# Supplementary material for: Interaction of Human Respiratory Syncytial Virus (HRSV) Matrix Protein with Resveratrol Shows Antiviral Effect
Source: Int J Mol Sci. 2024 Nov 28;25(23):12790. doi: 10.3390/ijms252312790 (PMC11640991; doi:10.3390/ijms252312790)
Supplement: Supplementary file 1 [file ijms-25-12790-s001.zip › ijms-3241054-supplementary.pdf]

Supplementary Material

Interaction of HRSV M protein with resveratrol shows antiviral effect

Thaina Rodrigues [1,2]; Jefferson de Souza Busso[1,2]; Rpahel Binicius Rodrigues Dias[1]; Isabella Otenio Lourenço[1,2]; Jessica Maróstica de Sa[1,2]; Sidney Jurado de Carvalho[1]; Icaro Putinhon Caruso[1,2*]; Fatima Pereira de Souza[1,2*]; Marcelo Andres Fossey[1,2*]

[1] São Paulo State University (UNESP), Department of Physics, Institute of Biosciences, Humanities and Exact Sciences, Rua Cristóvão Colombo, 2265, São José do Rio Preto, SP 15054-000, Brazil. e-mail: thaina.rodrigues@unesp.br (T.S.R); e-mail: jefferson.busso@unesp.br (J.S.B); e-mail: rvr.dias@unesp.br (R.V.R.D); e-mail: isabella.otenio@unesp.br (I.O.L); e-mail: jessica.marostica@unesp.br (J.M.S); email: sidney.carvalho@unesp.br (S.J.C) e-mail: icaro.caruso@unesp.br (I.P.C); e-mail: fatima.p.souza@unesp.br (F.P.S); e-mail: marcelo.fossey@unesp.br (M.A.F).

[2] Multiuser Center for Biomolecular Innovation (CMIB), São Paulo State University "Júlio de Mesquita Filho" (UNESP), São José do Rio Preto, SP, Brazil.

*Correspondences: e-mail: marcelo.fossey@unesp.br (M.A.F). fatima.p.souza@unesp.br, (F.P.S), icaro.caruso@unesp.br (I.P.C) Tel: +55 17 3221-2244.

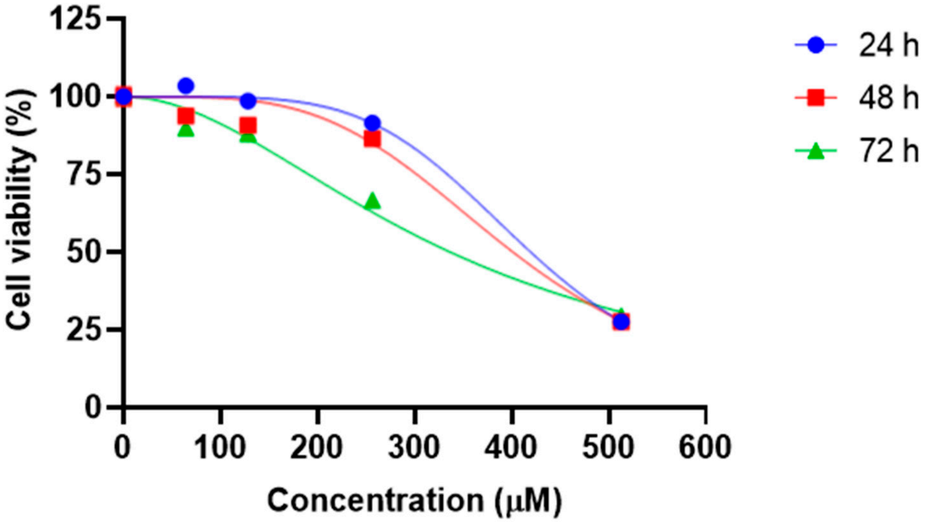

Figure S1. The figure shows the percentage of cell viability (x-axis) as a function of resveratrol concentration on a log scale (y-axis) at the different times tested. The blue circles represent 24h, the red squares represent 48h and the green triangles represent the 72h of cells in the presence of the compound.

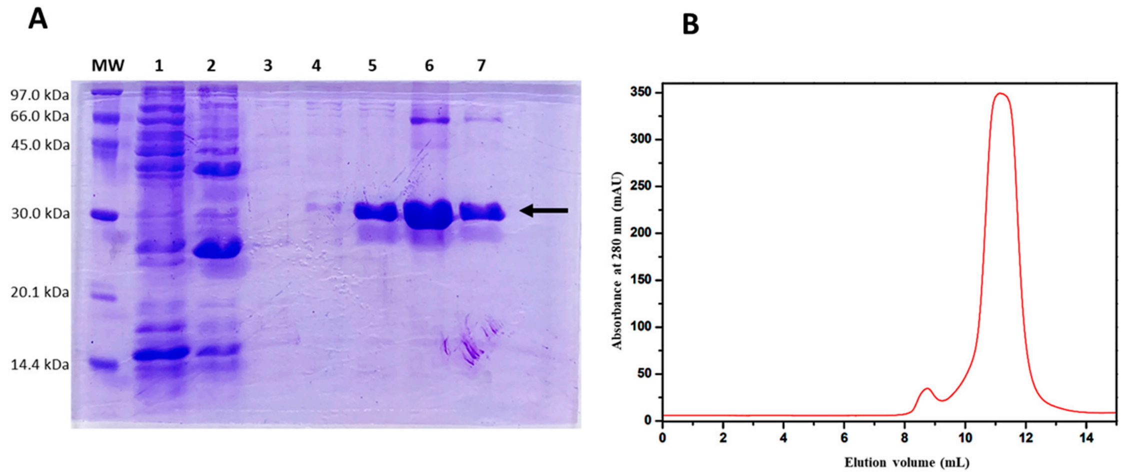

Figure S2. (A) SDS-PAGE stained with Coomassie Brilliant Blue R-250 showing the purification by affinity Ni+ chromatography. MW: molecular weight marker, Lane 1-4: washes with 5 to 40 mM Imidazole. Lane 5-7: 60 to 500mM imidazole with protein elution. (B) Size-exclusion chromatography of M protein with absorbance in 280 nm show the elution volume of the protein.

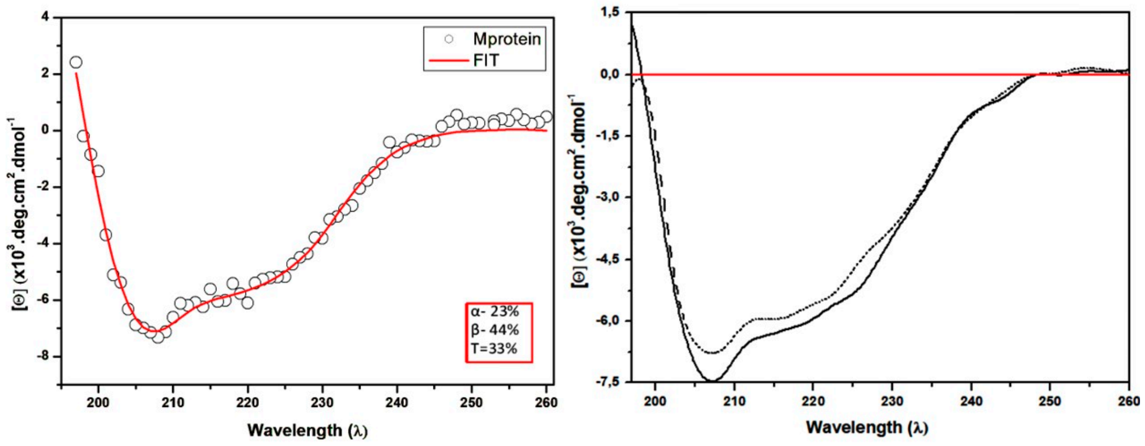

Figure S3. (A) Far CD-UV spectrum of M protein [5 μM] (empty black circles) at 298 K in buffer solution (150 mM Na_2HPO_4/NaH_2PO_4 pH 7.0, 50 mM NaCl, 1.0 mM BME). The red line represents

the best result for the fitted curve performed by the CONTINLL program. The box on the right represents the percentage of each secondary structure found, 23% of alpha helix structures, 44% of beta-strand, and 33% of random structures coil. (B) Far CD-UV spectrum of M protein [5 μM] (solid line) in phosphate buffer (150 mM Na2HPO4/NaH2PO4 pH 7.0, 50 mM NaCl, 1.0 mM BME) with the addition of Resveratrol [50 μM] (dashed line) with the buffer control (red line).

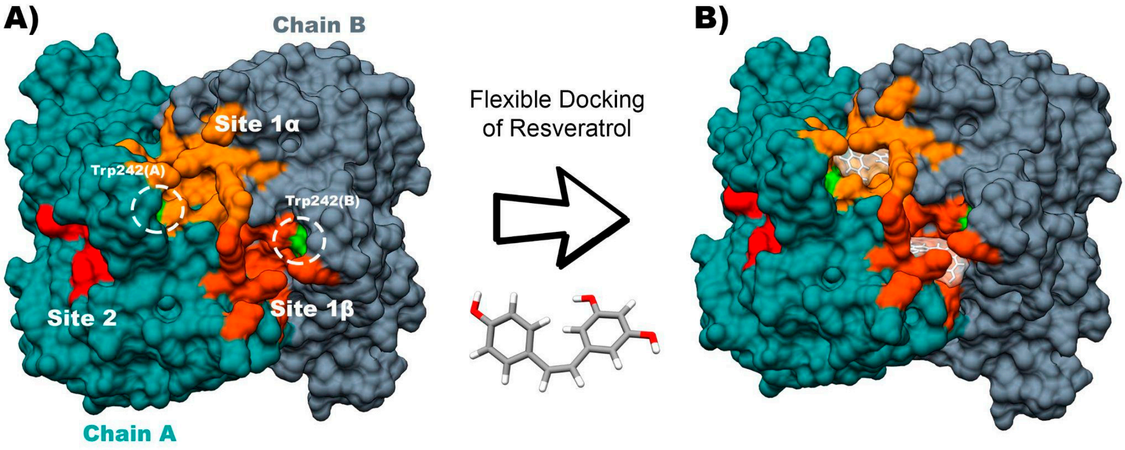

Figure S4. Site for interaction between M protein and Resveratrol. (A) The structure of the M protein, obtained from the PDB database (4V23), is represented in its molecular surface form, with chains A and B colored in cyan and gray to distinguish them. Highlighted are Site 1 (in shades of orange), represented by alpha and beta, as they are mirrored (symmetrical) sites, and Site 2 (in red). Tryptophan W242 is highlighted in green, as the presence of this residue is important for correlating with experimental fluorescence results. (B) The main anchor points of Resveratrol on the M protein show that the best fits are at Site 1, with no conformation of the ligand that matches Site 2. Resveratrol is represented in stick form and colored in white. Docking calculations were performed using the DOCK6 program, and the images were constructed and analyzed using the Chimera software.

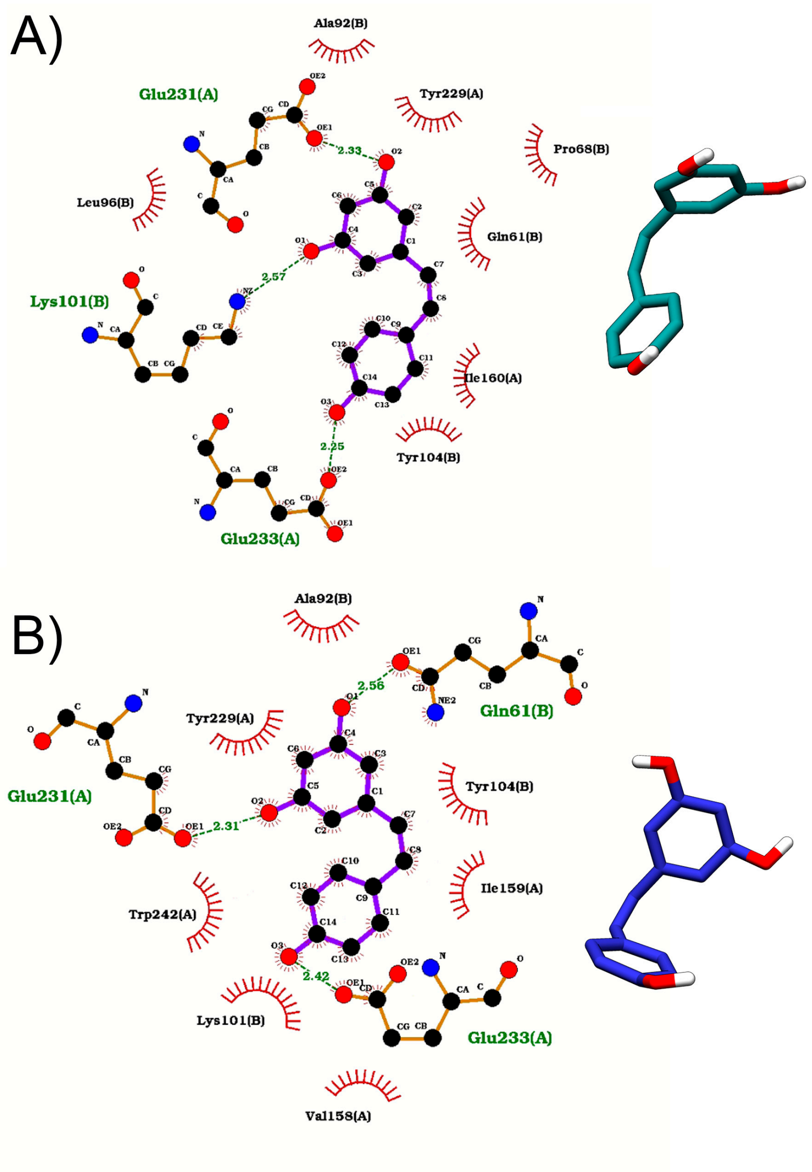

Figure S5. Conformations of Resveratrol at Site 1-Beta - The two conformations of resveratrol are similar, with a short difference in the A-ring structure. A) The most representative conformation observed during the molecular dynamics simulations displays three hydrogen bond interactions involving residues Lys 101, Glu 231, and Glu 233. Additionally, hydrophobic interactions are observed with the residues Gln 61, Pro 68, Ala 92, Leu 96, Tyr 104, Ile 160, and Tyr 229. B) In the second conformation, residues Glu 231 and Glu 233 continue to participate in hydrogen bonding, along with residues Ala 92, Lys 101, Tyr 104, Val 158, Tyr 229, and Trp 242. There is a notable similarity among the residues in both conformations and in comparison, to the other interaction site. The colors in the maps on the left indicate the types of interactions according to the software's design, while the molecules are colored only to differentiate conformation 1 (green) from conformation 2 (blue).
